# Effect of exposure to etidronic acid on the bond strength of calcium silicate-based cements after 1 and 21 days: an in vitro study

**DOI:** 10.1186/s12903-021-01959-5

**Published:** 2021-11-19

**Authors:** Elena Rebolloso de Barrio, Juan José Pérez-Higueras, Ernesto García-Barbero, Lucía Gancedo-Caravia

**Affiliations:** Department of Conservative and Prosthetic Dentistry, School of Dentistry, Complutense University, Pza. Ramon y Cajal, s/n. Ciudad Universitaria, 28040 Madrid, Spain

**Keywords:** Bond strength, Calcium silicate cements, Etidronic acid, Setting time, Root canal irrigants

## Abstract

**Background:**

After reparation of root perforations with calcium silicate-based cements (CSBC), the surface of the material is expected to be exposed to root canal irrigants (RCI) while resuming the root canal treatment.

**Methods:**

The aim of this study was to compare the effect of exposure to a mixture of sodium hypochlorite (NaOCl) and etidronic acid (HEBP) or other irrigants on the Push Out Bond Strength (POBS) of CSBC after two different setting times. 240 root slices 1 mm thick were obtained from single-rooted human teeth. A 1.4 mm diameter perforation was performed on each slice and filled with Biodentine (BD) or ProRoot MTA (PMTA). After 1 or 21 days they were exposed to 17% ethylenediaminetetraacetic acid, 5.25% NaOCl, a mixture of 5.25% NaOCl and 9% HEBP (NaOCl + HEBP) or saline (n = 15) and submitted to a push-out test. POBS results were analysed with ANOVA and Tukey tests.

**Results:**

BD showed higher POBS than PMTA after 1 day (p < .05). After 21 days no differences were found between materials. After 1 day exposure to NaOCl + HEBP resulted in higher POBS, compared to the other irrigants (p < .05).

**Conclusion:**

POBS results are influenced by the cement, the setting time and the exposure to irrigants.

## Background

Immediate root perforation repair is critical to avoid the contamination of the periodontal ligament, or the extrusion of root canal irrigants (RCI) [1] or root canal filling materials, which could cause an endodontic-periodontal lesion through damage to the epithelial attachment and bone loss [2]. For this reason, it is recommended to repair the perforation even before performing the root canal treatment [3].

An ideal repair material should, amongst other requirements, provide a suitable seal and have an adequate resistance to dislodgement under pressure caused by condensation forces applied to the restorative materials placed above it, or by occlusion loads [4].

Calcium silicate-based cements (CSBC) are commonly used to repair perforations [3, 5]. Mineral trioxide aggregate (MTA), and in particular ProRoot MTA (PMTA; Dentsply-Sirona, Ballaigues, Switzerland), was the first CSBC developed. Despite its widespread use, several drawbacks have been attributed to this cement, such as an extended setting time or discoloration of the tooth, among others [6]. To overcome these limitations, other materials have been developed. Biodentine (BD; Septodont, Saint-Maur-des-Fossés, France) is a CSBC that has shown a good performance in vitro and in vivo due to its biocompatibility, lower risk of tooth discoloration, shorter setting time and a similar ability to promote periradicular bone healing compared with PMTA [7–9]. The improved properties of BD in comparison to PMTA have been attributed to the differences in the composition of the two materials. The presence of bismuth oxide as a radiopacifier in MTA causes dentinal staining due to the reaction with collagen present in the organic dentin matrix [10] and it has also been reported to actively take part in the hydration reaction [11]. To prevent this problem, an inert component, zirconium oxide, has been added as a radiopacifier in BD [12, 13]. The PMTA powder is mixed with distilled water whereas the Biodentine liquid consists of an aqueous solution of calcium chloride (CaCl_2_), which acts as an accelerator of the hardening process, and a hydrosoluble polymer (water reducing agent) that allows good flowability with a low water/powder ratio [12, 14]. All these differences in the composition of the two cements may have an influence in their chemical reaction with other substances and, subsequently, in their physical properties.

After sealing the perforation, during cleaning and shaping of the root canal, the surface of the repairing material is expected to be exposed to different RCI, such as sodium hypochlorite (NaOCl), ethylenediaminetetraacetic acid (EDTA), or the recently introduced etidronic acid (also known as 1-hydroxyethylidene-1, 1-bisphosphonate, HEBP). HEBP is a biocompatible chelator that can be used in combination with NaOCl while maintaining the properties of both compounds [15]. Dual Rinse HEDP (MedCem, Vienna, Austria) has been developed as a HEBP powder to be mixed with sodium hypochlorite, which creates a stable solution to be used during the whole root canal treatment as a single irrigant [16, 17].

It has been established that contact with different RCI during setting time may influence the push-out bond strength (POBS) of CSBC [18–20], and this has been attributed to a variety of factors related to the compositions of the cements, or the properties of the RCI [19–21]. A number of studies have reported the deleterious effect of certain chelating agents on the properties of CSBC, and have attributed this to the interference of these substances with the setting reaction of the cements and with their chemical adhesion to dentine [19, 22–24]. However, until now, it has not been well established whether the exposure to the NaOCl + HEBP solution may affect the POBS. To our knowledge, only one study has recently tested the effect of exposure to NaOCl + HEBP on the POBS of several CSBC after 7 days of setting, concluding that after exposure to NaOCl + HEBP, BD obtained higher POBS values than with other RCI [25]. In addition, it has been observed that the POBS results of CSBC are conditioned by the time during which the cement is allowed to set before performing the pushout test [4, 26]. This influence of time may differ among cements as their setting times have been shown to be different [7]. Therefore, the clinical outcome of root perforations may be affected by the composition of the repairing materials, the properties of the RCI to which they are exposed during root canal treatment and the time elapsed between the placement of the material and the exposure.

The purpose of this study is to compare the effect of EDTA, NaOCl and NaOCl + HEBP on POBS of PMTA and BD in simulated root perforations after two different setting times (1 or 21 days). The following null hypotheses were tested:

- There is no difference in the POBS between cements, after exposure to different RCI or after different setting times.
- There is no difference in the failure pattern distribution among subgroups.

## Methods

### Specimen preparation

A total of 240 single-rooted freshly extracted human teeth were selected to be included in this study. All of them were evaluated by radiograph and under 10 × magnification (Leica MZ12 Leica Microsystem, Wetzlar, Germany). The selected teeth had single, non-calcified canals, and absence of radicular caries, resorptions or visible cracks. The soft tissue and the calcified debris of the radicular surface were removed with a manual scaler and then the teeth were stored in saline solution (0.9% Sodium Chloride Injection USP, B. Braun Medical S.A., Rubí, Barcelona, Spain) at 4ºC until further preparation.

**Fig. 1:**
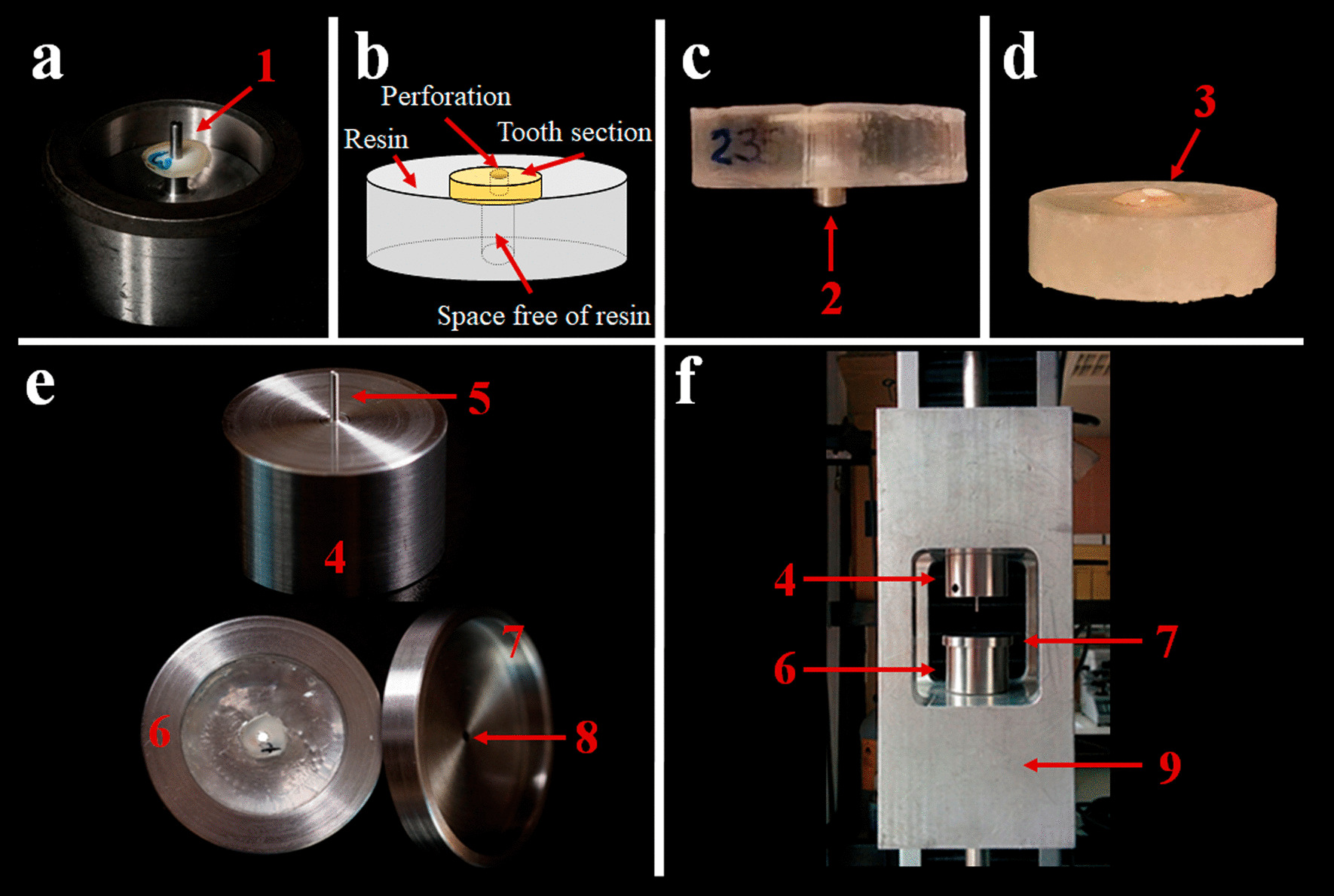
Sample preparation and POBS device. **a** Circular steel frame with a stem (1) to centre the dentin disc. **b** Schematic view of a specimen, consisting of a resin cylinder with the tooth slice on the upper surface and with the perforation centred and aligned with the axis. **c** Lateral view of the specimen with a metal cylinder (2) to prevent extrusion while filling the perforation. **d** Specimen with perforation filled with CSBC and covered with the RCI (3). **e** POBS device 4: upper piece holding the cylindrical punch (5), 6: lower piece—specimen holder, 7: cover, 8: hole to centre the punch during the test). **f** POBS device located in the testing machine (9: alignment device)

All the specimens were prepared as previously described [4, 25]. Shortly after the removal of the crown, a slice of 1 mm thickness was cut from the coronal end of every root. In the centre of the slice, a circular perforation of 1.4 mm diameter was drilled with a cylindrical diamond bur (Kerr C837-014, Bioggio, Switzerland). The slices were inserted into a circular metal frame, securing them through their perforation to a stem located in the centre of the frame to be embedded in resin (Activated Chronolite 1019, Plastiform, Cronolab, Madrid, Spain) (Fig. 1a). Once the resin had cured, the created specimens were extracted from the frames (Fig. 1b), obtaining cylindrical pieces with the tooth slice surrounded by chronolite, with the upper surface uncovered, and a lower perforation in the axis of the resin cylinder of 2.5 mm diameter and 5 mm height, aligned with the perforation of the root slice.

### Experimental groups

The specimens were randomly divided into 2 groups (n = 120), according to the repairing material used to fill the perforation: BD or PMTA. The cements were used following manufacturers’ instructions and placed inside the perforation of the specimens. To prevent the extrusion of the material, below the lower surface of the tooth a 2.5 mm diameter steel cylinder was inserted inside the lower perforation of the specimen (Fig. 1c). The excess material was removed from the upper surface with a scalpel. The specimens in each group were further divided into 2 subgroups according to the different setting times: 1 day, or 21 days. During setting, the specimens were stored at 37ºC and 95% humidity. Afterwards, the specimens were randomly divided to be exposed to 17% EDTA (EDTA, ENDO-SOLution, Cerkamed, Stalowa Wola, Poland), to 5.25% NaOCl (NaOCl, Chloraxid 5.25%, Cerkamed, Stalowa Wola, Poland), to a mixture of 5.25% NaOCl and 9% HEBP (NaOCl + HEBP, Dual Rinse HEDP) or remain stored in contact with a gauze wrapped in saline. An amount of 0.2 ml of RCI was applied on the coronal surface of the repairing material. The RCI was discarded and renewed every 5 min until the exposure time of 30 min was completed (Fig. 1d). The exposure time for EDTA was 5 min, and therefore no renewal was needed. In all, with this experimental design, we obtained a total of 16 groups (n = 15), based on the CSBC, the setting time and the RCI used. (See Table 1, in the results section).

### Push out bond strength test

The specimens were attached to an aligning device that held them centred below a 1.2 mm diameter cylindrical stainless-steel punch (Fig. 1e, f). Using a universal material testing machine (Hounsfield H-5000 M, Metrotec, Lezo, Spain), the punch was moved towards the repairing material at a constant crosshead speed of 1 mm/min. The maximal force (F) needed for the punch to dislodge the material was registered in Newtons, and the POBS was calculated in Megapascals using the following formula: POBS (MPa) = F(N) / S(mm^2^), where S is the contact surface between dentin and material, obtained as follows: S = 2 × r (mm) × π × h (mm) (where r is the radius of the perforation, π is the constant 3.14, and h is the thickness of the slice).

### Failure pattern analysis

The samples were assessed under 40 × magnification to record the pattern of failure: adhesive (between dentin and cement), cohesive (within the cement) or mixed failure (both patterns).

### Statistical analysis

For the POBS data, three-way ANOVA was used to detect interactions amongst the three independent factors (cement, setting time and exposure to RCI). Afterwards, the CSBC were compared and studied separately at each setting time, to explore the effect of the different RCI with one-way ANOVA. All the analyses were followed by applying Tukey’s multiple comparisons test. The failure patterns were analysed using a Chi-square test. The level of statistical significance was set at 0.05.

## Results

During the experimental process, 3 of the 240 specimens were discarded (PMTA-Saline-1D, BD-EDTA-1D and BD-Saline-21D groups) due to the friction of the punch against the dentin wall caused by an error in the location of the dentin slice in the aligning device.

**Fig. 2:**
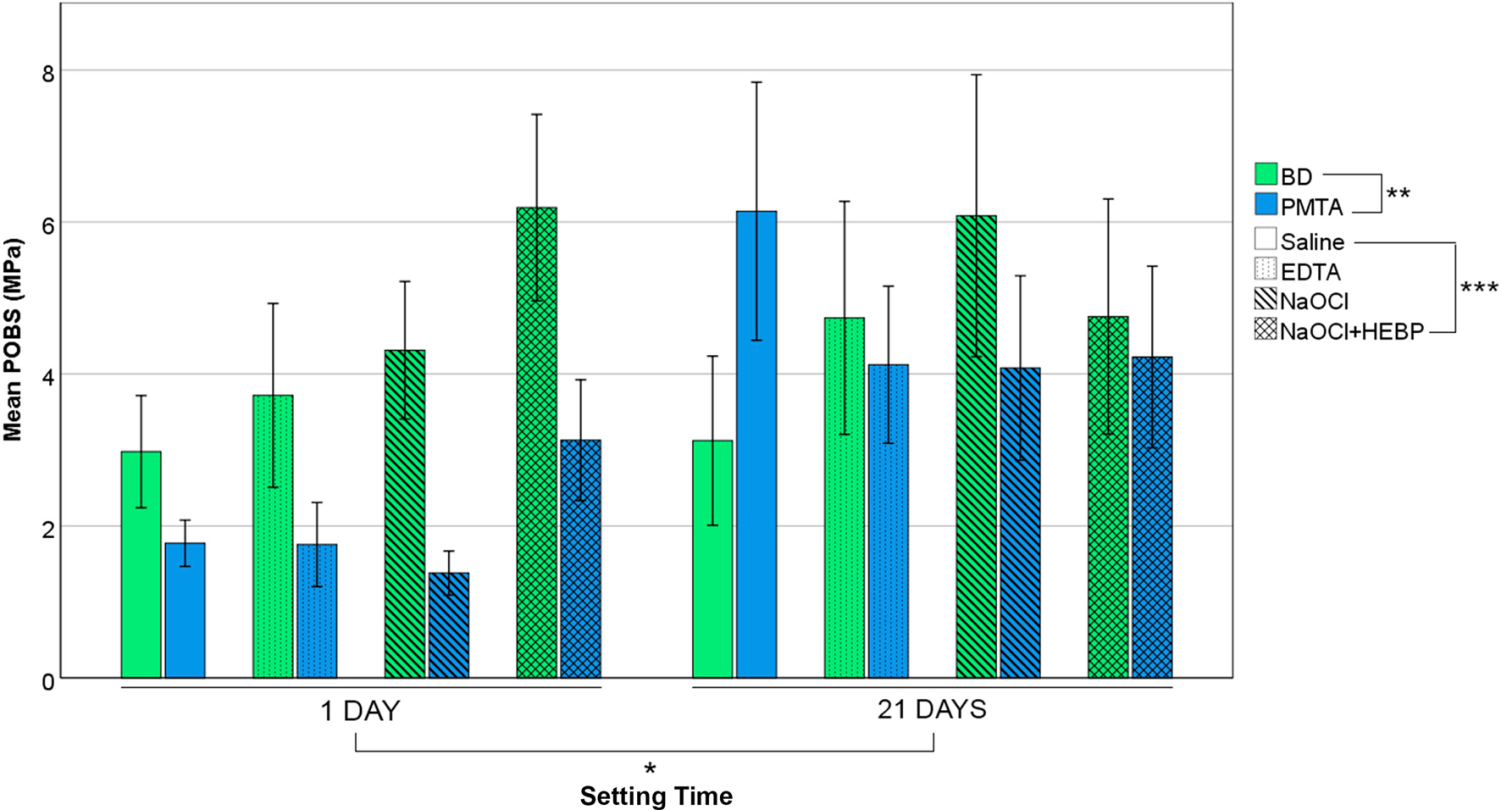
Bar Graph showing the mean POBS in MPa of each experimental subgroup. Error lines are calculated with a 95% confidence interval. BD, Biodentine; PMTA, ProRoot MTA; EDTA, Ethylenediaminetetraacetic acid; NaOCl, Sodium hypochlorite; HEBP, Etidronic Acid. Significant differences are represented as resulted from the Three-way ANOVA test among setting times (*), cements (**) and root canal irrigants (***)

The mean and standard deviation (SD) values (in MPa) of all experimental groups are shown in Table 1. Three-way ANOVA revealed that the POBS was significantly affected by cements (p < 0.001), setting time (p < 0.001) and exposure to RCI (p < 0.001). All double interactions were statistically significant (p < 0.001 for cement*setting time and cement*exposure to RCI, and p = 0.005 for setting time*exposure to RCI). BD showed higher POBS than PMTA after 1 day (p < 0.001). At 21 days, no differences were found between these cements. There were no statistically significant differences in BD POBS between 1 and 21 days of setting while PMTA showed higher POBS values after 21 days than after 1 day (p < 0.001). When separately analysing the effect of exposure to RCI on BD and PMTA after the two setting times, both BD and PMTA obtained higher POBS values when exposed to NaOCl + HEBP, compared to the other RCI after 1 day of setting (p < 0.001). No statistically significant differences among RCI were detected at 21 days for both BD and PMTA (Fig. 2).

**Table 1:** Mean and standard deviation (SD) of POBS values of the experimental groups

RCI	1 Day			21 Days			Total
	Biodentine	ProRoot MTA	Total	Biodentine	ProRoot MTA	Total	
	POBS (MPa)	POBS (MPa)	POBS (MPa)	POBS (MPa)	POBS (MPa)	POBS (MPa)	POBS (MPa)
	Mean (SD)	Mean (SD)	Mean (SD)	Mean (SD)	Mean (SD)	Mean (SD)	Mean (SD)
Saline	2.977 (1.332)^†^	1.772 (0.528)^†^	2.395 (1.180)	3.122 (1.927)^†^	6.141 (3.067)^†^	4.684 (2.964)	3.539 (2.516)
EDTA	3.718 (2.096)^†^	1.755 (0.999)^†^	2.703 (1.880)	4.738 (2.770)^†^	4.121 (1.866)^†^	4.429 (2.341)	3.581 (2.282)
NaOCl	4.312 (1.635)^†^	1.381 (0.523)^†^	2.846 (1.909)	6.083 (3.350)^†^	4.078 (2.191)^†^	5.081 (2.962)	3.963 (2.715)
NaOCl + HEBP	6.189 (2.215)^‡^	3.128 (1.436)^‡^	4.658 (2.405)	4.756 (2.793)^†^	4.224 (2.157)^†^	4.490 (2.466)	4.574 (2.417)
Total	4.309 (2.168)^*^	2.013 (1.151)	3.161 (2.077)	4.701 (2.893)	4.641 (2.463)	4.671 (2.674)	3.919 (2.507)
Biodentine							4.701 (1.618)
ProRoot MTA							3.338 (2.329)

RCI, root canal irrigant; EDTA, ethylenediaminetetraacetic acid; NaOCl, sodium hypochlorite; HEBP, etidronic acid

^†^^,‡^For the same cement and setting time, different symbols represent statistically significant differences in POBS values among RCI

^*^For the same setting time, significantly higher POBS between cements

**Fig. 3:**
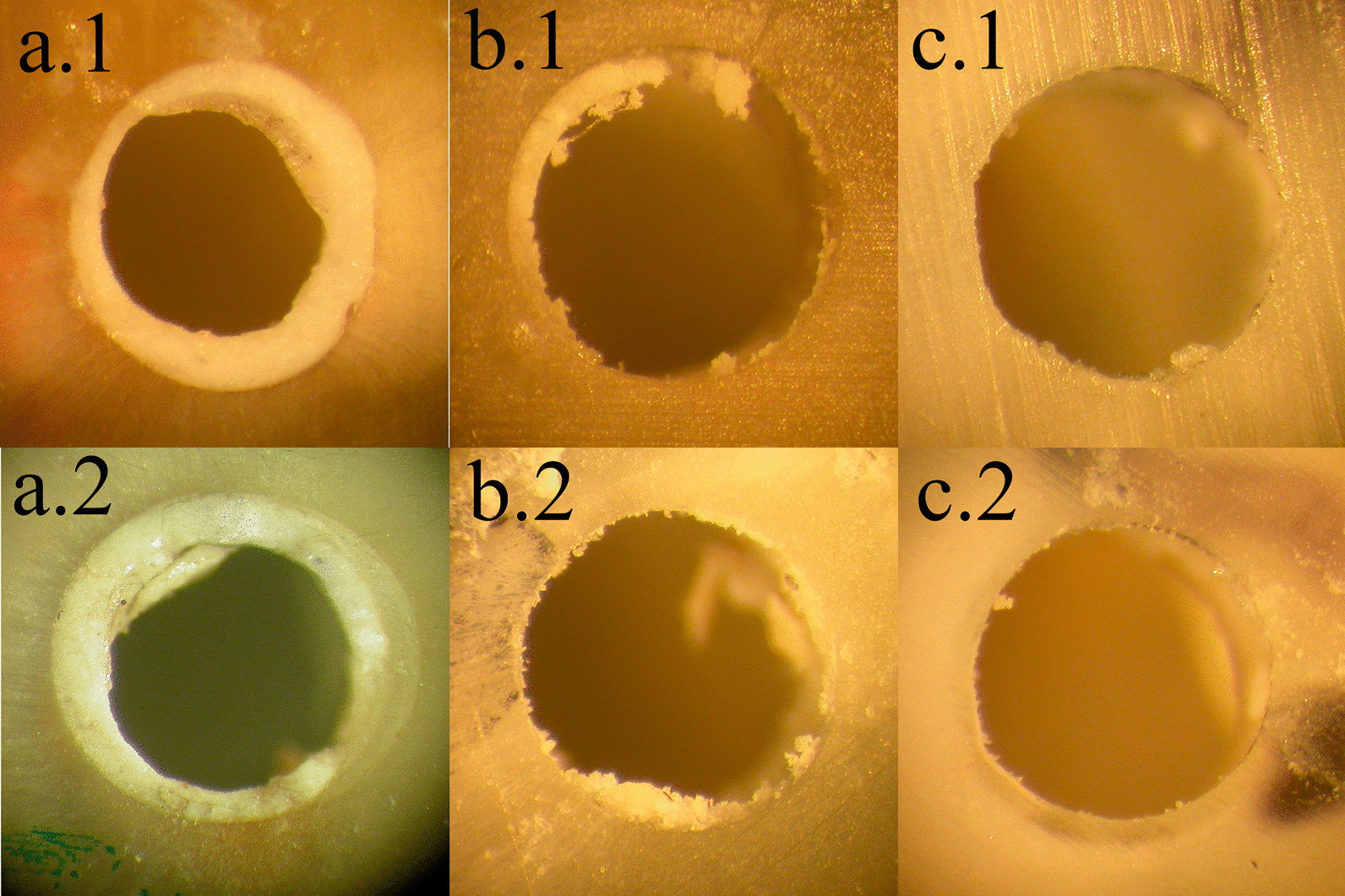
Representative stereomicroscope views at × 40 showing the different failure patterns. Cohesive failures in samples of BD-1 day-saline (**a.1**) and PMTA-21 days-NaOCl (**a.2**) groups; Mixed failures in samples of PMTA-1 day-NaOCl + HEBP (**b.1**) and BD-21 days-EDTA (**b.2**) groups; Adhesive failures in samples of BD-21 days-saline (**c.1**) and PMTA-1 day-NaOCl (**c.2**) groups

The total distribution of the different failure patterns in all the specimens was as follows: 35.4% cohesive, 38.8% mixed and 25.7% adhesive. A Chi-square test revealed no differences in the distribution of failure patterns among groups. The count and percentage of all the experimental groups are presented in Table 2. Examples of different failure patterns are shown in Fig. 3.

**Table 2:** Failure pattern distribution of the experimental groups

RCI	1 Day						21 Days					
	Biodentine			ProRoot MTA			Biodentine			ProRoot MTA		
	Failure patterns			Failure patterns			Failure patterns			Failure patterns		
	A	C	M	A	C	M	A	C	M	A	C	M
	c (%)	c (%)	c (%)	c (%)	c (%)	c (%)	c (%)	c (%)	c (%)	c (%)	c (%)	c (%)
Saline	6 (40.0)	3 (20.0)	6 (40.0)	5 (35.7)	5 (35.7)	4 (28.6)	5 (35.7)	2 (14.3)	7 (50.0)	2 (14.3)	9 (60.0)	4 (26.7)
EDTA	5 (35.7)	4 (28.6)	5 (35.7)	2 (13.3)	3 (20.0)	10 (66.7)	4 (26.7)	5 (33.3)	6 (40.0)	1 (6.7)	9 (60.0)	5 (33.3)
NaOCl	7 (46.7)	3 (20.0)	5 (33.3)	3 (20.0)	10 (66.7)	2 (13.3)	4 (26.7)	3 (20.0)	8 (53.3)	3 (20.0)	5 (33.3)	7 (46.7)
NaOCl + HEBP	3 (20.0)	6 (40.0)	6 (40.0)	1 (6.7)	9 (60.0)	5 (33.3)	4 (26.7)	3 (20.0)	8 (53.3)	6 (40.0)	5 (33.3)	4 (26.7)
Total	21 (35.6)	16 (27.1)	22 (37.3)	11 (18.6)	27 (45.8)	21 (35.6)	17 (28.8)	13 (22.0)	29 (52.3)	12 (20.0)	28 (46.7)	20 (33.3)

A, adhesive; C, cohesive; M, mixed; c, count; (%) percentage; RCI, root canal irrigant; EDTA, ethylenediaminetetraacetic acid; NaOCl, sodium hypochlorite; HEBP, etidronic acid

## Discussion

This research has reproduced the most frequently used parameters in POBS studies (i.e. the thickness of the slice, the size of the perforation or the incubation conditions of the specimens) with the aim of avoiding bias and allowing direct comparisons of the results among studies [27]*.* In order to create an accurate anatomical reproduction, Nagas et al. used a furcal perforation model [28], given that many of these perforations occur at the furcal level. Another design has been proposed to increase the reliability of the pushout test, which consists in placing different materials in perforations performed in the same dentin slice, to avoid the influence of individual variables of each tooth [29]. However, the use of radicular coronal third slices of uniradicular teeth with a single centred perforation may provide a more reproducible model and a more comparable situation among the tested materials. Decentred perforations may cause exposure to the tubules at different angles along the perimeter, which may have an influence on the penetration of CSBC in the dentinal tubules. Performance of the perforation centred in the root canal provides a homogeneous exposure of the dentinal tubules around the whole perimeter of the perforation.

Two setting times were established in order to compare the effect of RCI on the POBS values at different moments during the hydration process. The 21-day period of setting was selected to allow the complete maturation before testing, based on the results of a previously published research. In that study an increase in POBS of PMTA was observed between 1 and 21 days of setting when kept under wet conditions, suggesting a prolonged hydration process of this material [4].

Since our goal was to evaluate the effect of the tested irrigants on the POBS of repairing materials used to seal root perforations in simulated clinical conditions, exposure to the RCI was performed once the CSBC were placed in the perforation sites, instead of previously treating the dentin and then filling the perforations. Moreover, to extrapolate the results to clinical practice, the specimens were exposed to the RCI only on one side as previously described in the literature [30]. The exposure time for NaOCl was 30 min based on average reported clinical time of root canal preparation [18, 31]. A solution of 5.25% NaOCl was chosen for being one of the most commonly used in the clinical practice given that high concentrations have shown to be superior in terms of disinfection when compared to lower concentrations such as 1 or 2% [32, 33]. For NaOCl + HEBP the same exposure time as NaOCl was used as the authors that tested the properties of HEBP established that it can be used in combination with NaOCl as a single irrigating solution during the whole course of root canal preparation [15, 16]. EDTA was applied for a shorter time to reproduce usual protocols where it is used as a final rinse to remove the smear layer.

Our results showed that the POBS of BD was higher than that of PMTA. These findings agree with previous POBS studies [18, 26, 34]. Nevertheless, other authors have reported no differences between these repairing cements [35, 36]. The discrepancies in the results could be explained by the differences in the methodology among studies [27]. One of the differences in the study designs is the time elapsed between placement of the cement and the push-out test. In fact, when analysing the POBS results of the different subgroups of our study, we observed that the difference between BD and PMTA was only noticeable after 1 day of setting, while after 21 days, no difference between cements was detected. It has been reported that the POBS of MTA increases progressively with time, showing higher values after 21 or 28 days than after shorter times [4, 26], while BD has shown a much faster increase of its POBS, reaching high values after only 24 h [26]. This fact is in accordance with the results obtained after the two setting times in the present study. The rapid improvement of the mechanical properties of BD has been explained by the shorter setting time after mixing, due to the calcium carbonate contained in the BD powder that acts as a nucleation site for calcium carbonate hydrate and the addition of CaCl_2_ to the liquid as a setting reaction accelerator [12].

Regarding the role of RCI on the performance of the CSBC, the results of this study showed that contact with the tested RCI has different effects on the POBS of BD and PMTA. After 1 day, the exposure of BD and PMTA to NaOCl + HEBP solution obtained a higher POBS, compared to the exposure to the other RCI. After 21 days, no differences were found among RCI for any of the cements. Previous studies have reported that prior conditioning of dentin with a mixture of NaOCl and HEBP increased the POBS of calcium silicate-based materials [37, 38]. A recent study conducted with the same methodology as the present one tested the effect of exposure to NaOCl + HEBP and other RCI on the POBS of several CSBC after 7 days of setting, concluding that HEBP + NaOCl may improve the POBS values of BD and not those of PMTA [25]. This finding is in agreement with the results obtained in the present study after 1 day of setting for BD, however not with those obtained after 21 days. Another study has reported the high reactivity observed in a CSBC when exposed immediately after mixing to a mixture of NaOCl and HEBP for 30 min and described the formation of a highly crystalline surface [31]. All these observations suggest that the effect of this RCI on POBS could be related to the hydration process and the time that the cement needs to complete this process. It could also be associated with the specific chemical composition of each cement, such as the water soluble polymer or the CaCl_2_ added to the liquid of BD, which have been shown to improve the physical characteristics and the hydration process [39]. It has been hypothesised that the different radiopacifiers may play a role in the chemical reactions of the cements and therefore in their ultimate physical properties [11, 21, 40]. However, a clear explanation for the effect of NaOCl + HEBP on the CSBC has not been well established yet, as there is lack of knowledge about the actual chemical reaction of these substances.

In our study, exposure of the CSBC to different RCI did not influence their failure patterns. This is in accordance with other studies [18]. The higher prevalence of cohesive failure for BD has been widely reported in the literature [18, 37] while adhesive failure has been described as the most frequent type of failure for PMTA [30, 41]*.* In this study, no differences were found between cements.

Deeper understanding of the effect of RCI such as HEBP on the performance of CSBC is needed to establish treatment protocols and improve the outcome of perforation treatment in clinical situations. The present study was designed to test the POBS immediately after the exposure of the cements to RCI at two different setting times. Our results suggest that NaOCl + HEBP may be a suitable irrigant to be used shortly after repairing a perforation with CSBC as it has no detrimental effect on the bond strength of these materials. In fact, it seems to be beneficial when used after 24 h of setting. Nevertheless, further research focused on the effect of exposure time or on the role of the composition of CSBC or RCI could provide useful evidence for better handling of these materials.

## Conclusions

Based on the results of this study, it may be concluded that the POBS results are influenced by the cement, the setting time and the exposure to RCI. BD and PMTA exposed to NaOCl + HEBP after 1 day of setting obtained higher POBS than when exposed to other irrigants.

## Data Availability

The datasets used and/or analysed during the current study are available from the corresponding author on reasonable request.

## Abbreviations

BD: Biodentine
CSBC: Calcium silicate-based cements
EDTA: Ethylenediaminetetraacetic acid
HEBP: Etidronic acid (1-hydroxyethylidene-1, 1-bisphosphonate)
MTA: Mineral trioxide aggregate
NaOCl: Sodium hypochlorite
PMTA: ProRoot MTA
POBS: Push-out bond strength
RCI: Root canal irrigants
